# Supplementary material for: Spatiotemporal Dynamic Evolution and Its Driving Mechanism of Carbon Emissions in Hunan Province in the Last 20 Years
Source: Int J Environ Res Public Health. 2023 Feb 9;20(4):3062. doi: 10.3390/ijerph20043062 (PMC9965788; doi:10.3390/ijerph20043062)
Supplement: Supplementary file 1 [file ijerph-20-03062-s001.zip › ijerph-2135873-supplementary.pdf]

International Journal of
Environmental Research and Public Health

MDPI

Article

Spatiotemporal Dynamic Evolution and Its Driving Mechanism of Carbon Emissions in Hunan Province in the Last 20 Years

Huangling Gu [1,2,3], Yan Liu [2], Hao Xia [2], Xiao Tan [2], Yanjia Zeng [2] and Xianchao Zhao [2,3]*

1. Exploratory spatial-temporal data analysis (ESTDA)

Based on the traditional Exploratory Spatial Data Analysis (ESDA) framework, Rey et al. [1] effectively integrated time and space to achieve spatiotemporal interaction analysis, and proposed the ESTDA, which further improved the comprehensive process and spatiotemporal pattern analysis of geographic objects.

(1) LISA time path

The LISA time path is integrated to incorporate the dynamic migration of LISA coordinates in the Moran's *I* scatter plots. Through the pairwise movement of the attribute and spatial lag values of the carbon footprint of prefecture-level cities over time, the spatial-temporal interactions and dynamic characteristics of the carbon footprint at the local level within regions are thus explained. Expression of the local spatial dependence from the "instantaneous scene" to the "interactive dynamic scene" is continuously depicted [2]. The geometric characteristics of the LISA time path mainly include relative length (L_i), curvature (C_i) and average moving direction (γ_i), and their respective expressions are as follows [3]:

$$L_i = \frac{n \times \sum_{t=1}^{T-1} d(V_{i,t}, V_{i,t+1})}{\sum_{i=1}^{n} \sum_{t=1}^{T-1} d(V_{i,t}, V_{i,t+1})} \quad (1)$$

$$C_i = \frac{\sum_{t=1}^{T-1} d(V_{i,t}, V_{i,t+1})}{d(V_{i,t}, V_{i,t})} \quad (2)$$

$$\gamma_i = \arctan \frac{\sum_j \sin \gamma_j}{\sum_j \cos \gamma_j} \quad (3)$$

where *T* is the interval for the time series, *n* is the number of city-level units, and V_i, *t* is the LISA coordinate position (carbon emission standardization value) of city-level unit *i* in year *t*. In other words, (y_i, *t*, y_{V_i}, *t*), d(V_i, *t*, V_i, *t*+1) is the coordinate for the distance moved by city-level unit *i* in year *t* to *t*+1. If the length of the coordinate movement of city-level unit *i* is greater than the national average in the studied time range, then the city's L_i is greater than 1. The larger the L_i value, the more dynamic the local spatial dependence and spatial structure of the carbon footprint for city-level unit *i*, and vice versa. The larger the C_i, the more curved the LISA time path; namely, it is more affected by neighborhood space (spillover/polarization) in city *i*, and city *i* has a more volatile carbon footprint process and spatial dependent evolutionary process. In the opposite situation, the impact is less.

(2) LISA spatiotemporal transition

LISA first provides a local perspective to reveal the spatial dependencies between research units [4]. Rey then proposed the concept of spatial-temporal transition based on the transition of local spatial correlation types of each unit in Moran's *I* scatter plot in different periods [5]. The spatial-temporal transition of LISA reflects the transformation process of local spatial association types in the studied time range. There are four types of spatial-temporal transitions [6]. Type I is the diagonal type of transfer matrix, which means that there is neither spatial transfer within the city-level unit itself nor between the neighboring cities. Type II refers to a transition within the city-level unit itself, but the neighborhood is unchanged, including $HH_t \rightarrow LH_{t+1}$, $HL_t \rightarrow LL_{t+1}$, $LH_t \rightarrow HH_{t+1}$ and $LL_t \rightarrow HL_{t+1}$. Type III refers to no change within the city-level unit itself, but its neighborhood has a transition, including $HH_t \rightarrow HL_{t+1}$, $HL_t \rightarrow HH_{t+1}$, $LH_t \rightarrow LL_{t+1}$ and $LL_t \rightarrow LH_{t+1}$. Type IV refers to a transition within the city-level unit and in its neighborhood, divided into type IVA and type IVB. Type IVA refers to the transition direction of the city unit itself and its neighborhood are the same, including $HH_t \rightarrow LL_{t+1}$ and $LL_t \rightarrow HH_{t+1}$. Type IVB indicated that the transition direction of the city unit itself and its neighborhood are opposite, including $HL_t \rightarrow LH_{t+1}$ and $LH_t \rightarrow HL_{t+1}$. In addition, Rey et al. [7] defined the concepts of spatial-temporal flux (flux) and spatial-temporal cohesion (cohesion), whose formulas are as follows:

$$\text{flux:}\quad \mathrm{SF} = \frac{Y_2 + Y_3}{m} \quad (4)$$

$$\text{cohesion: } SC = \frac{Y_1 + Y_{4A}}{m} \quad (5)$$

where Y_1, Y_2, Y_3 and Y_4 represents the number of city-level units where the transition of Type I, Type II, Type III and Type IVA has occurred in the research time, respectively, while m is the total number of research units.

(3) Standard deviational ellipse (SDE)

SDE is a spatial statistical method to measure the distribution characteristics and spatial-temporal evolution of geographic elements [8]. The main parameters include distribution center of gravity, major axis (long axis), minor axis (short axis) and azimuth etc., which refer to the researcher of Chen et al. [9] for the detailed calculation process. The spatial distribution range of SDE represents the main area of the spatial distribution of geographic elements, the center of gravity represents the relative position of the spatial distribution of geographic elements, and the azimuth reflects the main trend direction of geographic element distribution (that is, the angle formed by the clockwise rotation of the due north direction to the long axis of the ellipse), and the long axis and short axis represent the degree of dispersion of geographic features in the primary and secondary directions, respectively.

2. GTWR model

The GTWR model adds the time dimension and considers the non-stationary characteristics of time and space, which makes the estimation more accurate, and it can reflect the spatiotemporal heterogeneity of different regions [10]. The basic formula is as follows:

$$Y_i = \beta_0(\mu_i, v_i, t_i) + \sum_k \beta_k (\mu_i, v_i, t_i) X_{it} + \varepsilon_i \quad (6)$$

where Y_i is the total carbon emission of city i; X_{ij} represents the value of city i for the k-th explanatory variable of the level of economic development, ecological environment, population, industrial structure, technological progress, per capita energy consumption, and land use are used as core explanatory variables. μ_i and vi are the longitude and latitude coordinates of city i, and t_i is the year t. $\beta_0(\mu_i,v_i,t_i)$ is the intercept term, $\beta_k(\mu_i,v_i,t_i)$ is the estimated coefficient of explanatory variable; ε_i is a random error term. The estimated value of each regression coefficient of city is:

$$\hat{\beta}(\mu_i, v_i, t_i) = \left[X^t W(\mu_i, v_i, t_i) X\right]^{-1} X^T W(\mu_i, v_i, t_i) Y \quad (7)$$

where $\hat{\beta}(\mu_i, v_i, t_i)$ is the estimated value of $\beta_k(\mu_i,v_i,t_i)$ and $W(\mu_i,v_i,t_i)$ is the space-time weight matrix. X is the matrix formed by independent variables; X^t is the transpose of the matrix; Y is the matrix of observed values. In order to avoid the "long tail effect" caused by data discreteness, this study combined the distance threshold method and Gaussian function method commonly used in weight determination, and adopted the finite Gaussian function, namely b_i-square spatial weight function. The spatiotemporal distance between sample i and sample j is:

$$d_{ij} = \sqrt{\delta\left[\left(U_i - u_j\right)^2 + \left(v_i - v_j\right)^2 + \mu\left(t_i - t_j\right)^2\right]} \quad (8)$$

In Formula (8), Bandwidth B will affect the establishment of the space-time weight matrix. Considering the density of the data observation point distribution, adaptive bandwidth is adopted in this study, and the established criterion is AICc criterion.

References

1. Rey, S.-J.; Janikas, M.V. STARS: Space-time analysis of regional systems. *Geographical Analysis* **2006**, 38, 67-86. doi: 10.1111/j.0016-7363.2005.00675.x.
2. Ye, X.-Y.; Rey, S.-J. A framework for exploratory space-time analysis of economic data. *The Annals of Regional Science* **2013**, 50, 315-339. doi: 10.1007/s00168-011-0470-4.
3. Rey, S.-J. Space–time patterns of rank concordance: Local indicators of mobility association with application to spatial income inequality dynamics. *Annals of the American Association of Geographers* **2016**, 106, 788-803. doi: 10.1080/24694452.2016.1151336.
4. Anselin, L. Local indicators of spatial association—LISA. *Geogr. Anal.* **1995**, 27, 93-115. doi: 10.1111/j.1538-4632.1995.tb00338.x.
5. Rey, S.-J. Spatial empirics for economic growth and convergence. *Geogr. Anal.* **2001**, 33, 195-214. doi: 10.1111/j.1538-4632.2001.tb00444.x.
6. Rey, S.-J.; Murray, A.T.; Anselin, L. Visualizing regional income distribution dynamics. *Letters in Spatial and Resource Sciences* **2011**, 4, 81-90. doi: 10.1007/s12076-010-0048-2.

7. Ye, X.-Y.; Carroll, M.-C. Exploratory space-time analysis of local economic development. *Applied Geography* **2011**, 31, 1049-1058. doi: 10.1016/j.apgeog.2011.02.003.
8. Shi, Y.; Matsunaga, T.; Yamaguchi, Y.; Zhao, A.; Li, Z.; Gu, X. Long-term trends and spatial patterns of PM2.5-induced premature mortality in South and Southeast Asia from 1999 to 2014. *Sci. Total Environ.* **2018**, 631, 1504-1514. doi: 10.1016/j.scitotenv.2018.03.146.
9. Chen, L.; Xu, L.-Y.; Cai, Y.-P.; Yang, Z.-F. Spatiotemporal patterns of industrial carbon emissions at the city level. *Resour. Conserv. Recy.* **2021**, 169, 105499. doi: 10.1016/j.resconrec.2021.105499.
10. Huang, B.; Wu, B.; Barry, M. Geographically and temporally weighted regression for modeling spatio-temporal variation in house prices. *Int. J. Geogr. Inf. Sci.* **2010**, 24, 383-401. doi: 10.1080/13658810802672469.
